# Identification of Anomalies in Mammograms through Internet of Medical Things (IoMT) Diagnosis System

**DOI:** 10.1155/2022/1100775

**Published:** 2022-09-22

**Authors:** Amjad Rehman Khan, Tanzila Saba, Tariq Sadad, Haitham Nobanee, Saeed Ali Bahaj

**Affiliations:** ^1^Artificial Intelligence & Data Analytics Lab CCIS, Prince Sultan University, Riyadh 11586, Saudi Arabia; ^2^Department of Computer Science and Software Engineering, International Islamic University, Islamabad 44000, Pakistan; ^3^College of Business, Abu Dhabi University, Abu Dhabi, UAE; ^4^Oxford Center for Islamic Studies, University of Oxford, Oxford, UK; ^5^Faculty of Humanities & Social Sciences, University of Liverpool, Liverpool, UK; ^6^MIS Department College of Business Administration, Prince Sattam Bin Abdulaziz University, Alkharj 11942, Saudi Arabia

## Abstract

Breast cancer is the primary health issue that women may face at some point in their lifetime. This may lead to death in severe cases. A mammography procedure is used for finding suspicious masses in the breast. Teleradiology is employed for online treatment and diagnostics processes due to the unavailability and shortage of trained radiologists in backward and remote areas. The availability of online radiologists is uncertain due to inadequate network coverage in rural areas. In such circumstances, the Computer-Aided Diagnosis (CAD) framework is useful for identifying breast abnormalities without expert radiologists. This research presents a decision-making system based on IoMT (Internet of Medical Things) to identify breast anomalies. The proposed technique encompasses the region growing algorithm to segment tumor that extracts suspicious part. Then, texture and shape-based features are employed to characterize breast lesions. The extracted features include first and second-order statistics, center-symmetric local binary pattern (CS-LBP), a histogram of oriented gradients (HOG), and shape-based techniques used to obtain various features from the mammograms. Finally, a fusion of machine learning algorithms including K-Nearest Neighbor (KNN), Support Vector Machine (SVM), and Linear Discriminant Analysis (LDA are employed to classify breast cancer using composite feature vectors. The experimental results exhibit the proposed framework's efficacy that separates the cancerous lesions from the benign ones using 10-fold cross-validations. The accuracy, sensitivity, and specificity attained are 96.3%, 94.1%, and 98.2%, respectively, through shape-based features from the MIAS database. Finally, this research contributes a model with the ability for earlier and improved accuracy of breast tumor detection.

## 1. Introduction

The cells in the body that tend to change their natural form properties cause cancer. Breast cancer is a widespread syndrome in females around the globe. According to ACS (American Cancer Society), around 42,170 females expired due to breast malignancy in the USA in 2020 [1]. This report reveals breast cancer's second major cause of demise among females. The chances of breast cancer and death rates usually tend to increase with age. But it is also observed that the survival rate is much higher if cancer is found only in the breast part of the body. Thus, an early finding of breast malignancy is significant for survival. Usually, mammography is considered a highly reliable and low-cost procedure for early breast cancer diagnosis [2]. Modern screening procedure of mammography used in Canada and Europe reveals that approximately 40% chance of death due to breast cancer is reduced among females that are exposed [3]. To lessen the workload of radiologists, CAD system was brought into action. It is utilized as a “second opinion” for radiologists to investigate anomalies from mammogram images through computational methods [4]. Various studies have suggested that a cloud-based CAD system benefits patients in remote and rural areas, especially in breast cancer [5]. The development of smart technologies created our everyday life more comfortable than earlier. The innovation of the IoMT devices and the cloud services provided another direction for the system's effectiveness. The E-health care systems allow specialists to examine the patient data remotely with minimum cost [6, 7]. There are several other reasons for implementing E-health care systems to find breast malignancy, such as lack of specialist radiologists.

Pakistan is an underdeveloped country and most of the population resides in rural areas, where no proper arrangement of health services is made. Similarly, lack of education is another reason females are unaware of their health conditions. Thus, it is essential to cover the disparity between urban and rural areas. For this reason, teleradiology has been employed to assist rural communities and overcome the distance barriers as far as medical services [8]. In rural and remote areas, connectivity issues frequently occur and limit the advantages of mobile mammography devices. In such environments, a CAD system is a sound that could be deployed with the mammography device to detect breast anomalies with or without human interference.

## 2. Background

Several researchers have proposed breast tumor detection using different techniques based on texture and shape features [9, 10]. The idea of the E-healthcare system is also emerging with time. The following section provides detailed literature about breast tumor classifications, detection, and the E-healthcare system.

### 2.1. Breast Tumor Detection and Classifications

An assortment of CAD systems is discussed in the literature. Most of the systems vary in terms of extracting features from the imageries. A concise review of preprocessing, segmentation methods, and texture-based feature extraction procedures of CAD are highlighted in this portion. In the preprocessing stage, the artifacts in the mammogram images, including noise and pectoral muscles, are eliminated. The presence of such factors can stimulate the extraction of imprecise features that may cause misclassification [4]. The cropping strategy has been found proficient in numerous studies to expel unwanted parts and acquire the region of interest (ROI). The ROI is chosen by taking the expected circle's radius encompassing the tumor part.

Segmentation is the most crucial phase of a CAD system and essential features are retained through efficient segmentation. The approaches used for segmentation can generally be partitioned into two groups: edge-based and region-based techniques. Edge-based techniques look for discontinuity in an image region and region-based splits an image into regions in the fashion that all resultant regions become similar where each contains at least one common property or feature (such as surface, reflectivity, shading, color, and so forth) [11, 12]. Usually, the region growing [13], FCM [14], and K-means [15] algorithms are used for breast tumor localization.

The feature extraction step is accepted as another significant part of CAD systems. A variety of frameworks is assembled for this procedure. Grey-Level Co-occurrence Matrix (GLCM) is a well-known scheme applied for feature extraction. Several studies proposed the GLCM technique to define texture-based features in the mammogram CAD system [16]. LBP is another unique texture extraction mechanism that separates benign masses from malignant ones. However, the recognition speed of LBP is slowed down due to long histograms and also missed actual intensity levels during computation at some point. Thus, advancements in LBP like LBP Variance (LBPV) and Completed LBP (CLBP) descriptors are applied to solve the problems of LBP [17].

Lahoura et al. [2] proposed a cloud computing-based model for remote breast cancer detection and used ELM (variants of ANN) to diagnose breast cancer on the Wisconsin Diagnostic Breast Cancer (WBCD) dataset. The accuracy, recall, precision, and F1-score were claimed at 98.68%, 91.30%, 90.54%, and 81.29%. Shao et al. [18] used S-WAVE data to classify breast cancer in 40 patients. Bi-spectral and Wigner spectrum features were extracted and classified using fusion of Random Forest and Support Vector Machine. Different feature sets have been experimented and the highest accuracy of 95% is reported. Sadad et al. [19] automatically identified breast density (BD) from mammogram images utilizing IoMT environments. Two deep convolutional neural network models, DenseNet201 and ResNet50, were implemented using a transfer learning strategy. The Mammogram Image Analysis Society collection had a total of 322 mammogram images, including 106 fatty, 112 dense, and 104 glandular instances. The DensNet201 model achieved a classification accuracy of 90.47% for BD detection. Hamed et al. [20] performed breast cancer recognition using You Only Look Once (YOLO) and RetinaNet models and attained 91% accuracy. However, no benchmark datasets were experimented. Li et al. [21] integrated the highly interconnected UNet (DenseNet) with Attention Gates (AG). The model was trained using the cross-entropy loss function and claimed 82.24%, 77.89%, and 78.38% of F1-score, sensitivity, and accuracy. Saba et al. [22] produced a cloud-based decision support system for the identification and categorization of malignant cells in breast cancer by extracting shape-based information from a breast cytology images. To identify breast cancer, naive Bayesian and artificial neural networks are used and 98% accuracy is reported. However, they did not use benchmark datasets and medium-size local dataset experimented.

Finally, a summary of related work on breast cancer diagnosis is presented in Table 1.

### 2.2. Mobile Devices for E-Healthcare

Cloud computing is necessary for E-healthcare systems because computational power, storage space, and network bandwidth are often needed in most image processing methodologies [24, 25]. Literature reveals that there are numerous cloud-based systems available for E-healthcare. The purpose of such models is to use precious resources efficiently. Currently, the idea of IoMT is employed mainly for remote patient monitoring. Therefore, efficient machine learning models are still highly demanded practical image analyses and cancer diagnosis remotely [26].

The main contributions of the proposed model are summarized below.

- IoMT-based decision support architecture for remotely identifying breast tumors and the quality of healthcare facilities is proposed.
- A region-growing algorithm for mammography tumor segmentation is implemented.
- Explored novel textural analysis techniques to precisely describe segmented tumours, i.e., First and Second-Order Statistics features, CS-LBP, HOG, and Shape-based features extraction methods are applied to segmented masses to obtain the powerful features.
- Exhibited high classification performance using sensitivity, specificity, accuracy, and MCC and achieved higher classification accuracy.

The rest of the study is structured as such: Section 3 exhibits proposed design of IoMT mammography unit.  Section 4 explores the proposed CAD system. Results and discussions are presented in Section 5 and finally, research is concluded in Section 6.

## 3. Proposed Method for IoMT Mammography Unit

**Figure 1:**
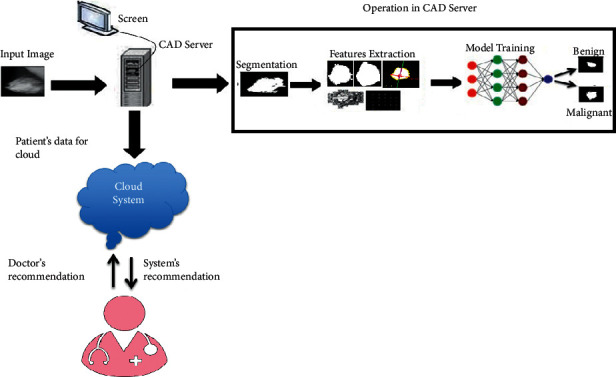
Proposed research framework.

A cloud-based approach for mobile devices is an emerging technique for the E-health care system [27]. The proposed research model is presented in Figure 1. In the proposed method, the operation of CAD system for the classification and detection of the tumor can be performed locally. Then, the decision of the CAD system and the mammography images are uploaded to the cloud. The radiologists can retrieve the images and diagnostic results (obtained through CAD system) from anywhere and anytime by using their credentials to verify whether the case is benign or malignant. Integration of CAD system with mobile mammography unit provides an initial diagnosis of breast abnormality if present. This finding will provide an early intimation to the patient because there is always a delay in obtaining investigation reports due to a lack of expert radiologists in remote areas [28]. Tele-radiology through IoMT mammography machine can be used in areas with a deficiency of medical experts. Thus, implementing a CAD system with a mobile mammography system will facilitate computer radiographers to judge the tumor status and further inform the radiologist/physician through the cloud system about abnormality.

## 4. Proposed Method for IoMT Mammography Images

**Figure 2:**
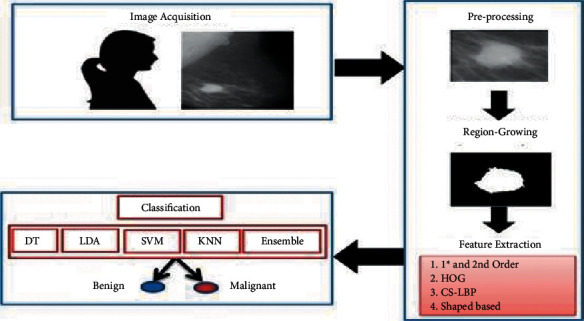
Proposed CAD system.

In this section, the proposed system CAD for breast malignancy has been explained. The proposed system comprises preprocessing, segmentation features extraction, and classification of masses as depicted in Figure 2.

### 4.1. Dataset

The Mammographic Image Analysis Society (MIAS) dataset has experimented in this research and made accessible online [29]. This database encompasses 322 mammogram images of 161 patients and provides comprehensive information about the position, severity, and abnormalities. The purpose of employing this database is to comprise mammogram imageries with a high noise level, making the task of lesion classification very tough [30].

### 4.2. Preprocessing

It is crucial and is exercised before any procedure to achieve the appropriate accuracy [31]. The mammographic images encompass different sorts of noise and also include pectoral muscles. So, the concentration is to expel undesirable areas such as pectoral muscles, labels, etc. These undesirable components can enforce the extraction of inaccurate features, leading to misclassification. A cropping technique is utilized to dispense the undesirable segments and get regions of interest (ROIs). This procedure begins from the center of the tumor part and takes the approximated radius around the tumor region [9, 13]. The mammogram images are cropped according to the MIAS dataset's parameters to attain ROI.

### 4.3. Segmentation

Image segmentation is a core part of tumor detection in medical imaging analysis [32]. During mammogram image segmentation, the parts of the tumor are separated from the background tissues to detect masses, micro-calcifications, and speculated lesions. An assortment of techniques is available for segmentation. Still, none of those can produce consistent performance. Not every method is universally accepted for all images because every imaging technology and procedure have specific limitations [33]. The region growing method is used for tumor segmentation in the proposed technique.

This technique groups pixels into regions built on a pre-defined growing principle. The growing starts with a seed point or a set of seed points and the region is iteratively grown by adding nearby elements to the seed point having similar properties. This procedure continues until no further adjacent pixels satisfying the growing condition are left [34].

The following steps are performed for region-growing segmentation:

- Input image = ROI;
- Select the seed point;
- Ensure the adjacent elements and append them to the area similar to the seed point;
- Reiterate step (iii) for more newly elements;
- Discontinue if no further points can be added;

### 4.4. Feature Extraction

It presents a means to transform an image array into informative dimensions for the consequent phase. Texture features are the standard extraction methods of a CAD system. In this CAD system, different sorts of feature extraction are First and Second-order Statistics features, CS-LBP, HOG, and Shape-based features.

#### 4.4.1. First and Second-Order Statistics Features

Statistical approaches consider the spatial distribution of gray levels by calculating local characteristics at every object element. Statistical methods may be further categorized into first-order, second-order, and higher-order statistics representing one pixel, two pixels, and three or more pixels to describe the local features [35]. The first-order statistics estimate the pixel characteristics (e.g., mean and variance); ignoring the spatial relationship. While second-order statistics evaluate the relationship among two values of pixel happening at particular positions. The higher-order statistics (considering the relationships among three or more pixels) are theoretically possible but not implemented practically due to interpretation and complexity in calculation time.

First-order statistics-based approach: The image's pixel values, such as mean, were considered first-order texture features. The histogram-based approach is founded on the gray level distribution on all or part of an image [35]. The first-order histogram estimate *P*(*v*_*i*_) is calculated as depicted in (1) [36].

(1) $$\begin{array}{c} P\left(v_{i}\right)=\frac{N\left(v_{i}\right)}{S}\text{,} \end{array}$$

*N*(*v*_*i*_)is the number of pixels used to represent gray levels in the object, *S* is the size of the image, *i*=0, 1, 2, 3,……., *L* − 1 and *L* are distinct gray levels of the histogram. The feature extraction from the histogram is used in this work, including Energy, Mean, Variance, Entropy, Skewness, and Kurtosis.

Mean: The mean provides the average gray level of each region, as shown in equation (2).
  (2) $$\begin{array}{c} Mean=\sum_{i=0}^{L-1}v_{i}P\left(v_{i}\right)\text{.} \end{array}$$
*Variance*: Several grey-level variations from the mean are presented in equation (3).
  (3) $$\begin{array}{c} Variance=\sum_{i=0}^{L-1}{\left(v_{i}-m\right)}^{2}P\left(v_{i}\right)\text{.} \end{array}$$
*Skewness* is an amount of the gray levels' asymmetry around the mean as shown in equation (4).
  (4) $$\begin{array}{c} Skewness=\sum_{i=0}^{L-1}{\left(v_{i}-m\right)}^{3}P\left(v_{i}\right)\text{.} \end{array}$$
*Kurtosis*: It describes the histogram sharpness as illustrated in equation (5).
  (5) $$\begin{array}{c} Kurtosis=\sum_{i=0}^{L-1}{\left(v_{i}-m\right)}^{4}P\left(v_{i}\right)\text{.} \end{array}$$
*Energy*: Energy describes the homogeneity of the texture. So, the energy will be high when its value is uniform and vice versa, as shown in equation (6).
  (6) $$\begin{array}{c} Energy=\sum_{i=0}^{L-1}v_{i}P{\left(v_{i}\right)}^{2}\text{.} \end{array}$$
*Entropy*: It describes the uncertainty of gray value distribution. So, the entropy will be high when gray values are randomly distributed in the image, as presented in equation (7).
  (7) $$\begin{array}{c} Entropy=-\overset{L-1}{\underset{i=0}{\sum }}v_{i}\log_{2}\;P\left(v_{i}\right)\text{.} \end{array}$$

Second-order statistics-based approach: The features can be obtained from the co-occurrence that assesses the image's properties associated with second-order statistics. The relationships among pixels are considered in pair [37]. Usually, the co-occurrence matrix is processed based on two parameters: relative distance among the pixel pair and their relative orientation. The orientation *θ* is approximated in four different directions, which are *θ*=[0°, 45°, 90°, 135°] horizontal, diagonal, vertical and anti-diagonal respectively. For every feature, four values were calculated according to the four directions of *θ*. The average of these four values is estimated to obtain 7 features of GLCM, including Correlation, Inertia, Inverse Difference, Angular second Moment, Absolute Value, Maximum Probability, and Entropy.

#### 4.4.2. Histogram of Oriented Gradient (HOG)

HOG is a robust descriptor that denotes gradient orientation, i.e., the angle and magnitude of an image [38]. In HOG, the local object's characteristics (shape and appearance) are described via the distribution of edge directions or intensity gradients. The operation to obtain shape and appearance can be accomplished by splitting the object into cells and computing a histogram of each cell's edge orientations or gradient directions. The combination of these histograms obtains the descriptor. It is suitable for better shadowing and outcome to contrast-normalize the local responses before employing them. Such achievement is achieved by calculating the intensity through a larger region (“blocks”), then utilizing this value to normalize all cells in the block. The normalized descriptor blocks are known as HOG descriptors.*(1) Algorithm Implementation*: The HOG is computed in the following three steps.

Step 1 (*Gradient Computation)*: The first phase calculates the gradient values. This is done in two stages. The first stage of gradient computation calculates a centered mask to smoothen the intensity data or color of the image. The most well-known technique to achieve this goal is to apply a 1-D derivative mask [−1, 0, +1] and [−1, 0, +1]^*T*^ in one or both of the vertical and horizontal directions. The second stage of gradient computation is to compute the gradient magnitude (*u*, *v*)and gradient angle *θ*(*u*, *v*)utilizing *u*- and *v*-directional gradients ∇*u*(*u*, *v*)and ∇*v*(*u*, *v*) for every pixel in a cell.
  (8) $$\begin{array}{c} m\left(u,v\right)=\sqrt{\nabla u{\left(u,v\right)}^{2}+\nabla v{\left(u,v\right)}^{2}}\text{,}\\ \theta =\arctan\frac{\nabla v\left(u,v\right)}{\nabla u\left(u,v\right)}\text{.} \end{array}$$
Step 2 (*Orientation Binning)*: The orientation binning process makes the cell histograms. Every pixel in the cell emits a weighted vote for a direction binning on the first step's values (i.e. Gradient Computation). For example, the cells could be radial or rectangular, and orientation bins are distributed equally over 0 to 180° (“unsigned” gradient) or 0 to 360° (“signed” gradient). Higher magnitude values are considered part of edge directions while lighter magnitude values are disposed of. A 9-bin histogram relating to every pixel orientation is formed using weighted magnitude for each cell.
Step 3 (*Descriptor Blocks)*: Features are extracted from every cell and cells are linked to each other to build a block descriptor. Finally, a final descriptor of a block is created after concatenating the histograms of cells.

#### 4.4.3. Center-Symmetric Local Binary Pattern (CS-LBP)

**Figure 3:**
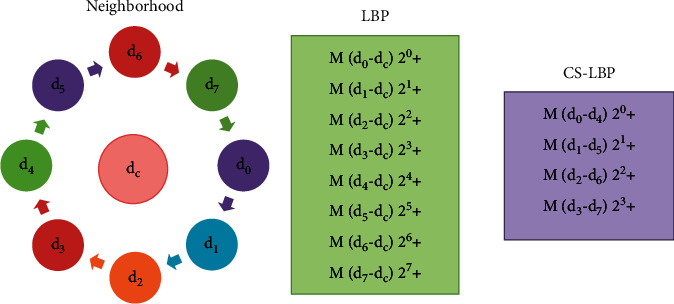
LBP and CS-LBP features comparison.

The CS-LBP is an improved form of the LBP and Scale-Invariant Feature Transform (SIFT) descriptor to get the appropriate features (based on texture and gradient). In CS-LBP, the extractions of features are like that of the LBP operator and the creation of the descriptor is the same as in SIFT. This approach comprises several properties: robustness, computational simplicity, and tolerance to illumination changes [39]. Heikkilä et al. [40] express that a long histogram is created in the LBP, making it hard to employ it for an image descriptor. A review for comparing the neighborhood pixels with the center one is needed to resolve this dilemma. As a substitute, the CS-LBP approach compares symmetric center pairs of pixels as shown in Figure 3. As it can be seen that for 8 adjacent points, LBP creates 256 distinctive binary values while CS-LBP delivers just 16 different binary patterns. To create the descriptor, the ROI image is split into cells with a location grid and a histogram of CS-LBP is then constructed for each cell. The feature extraction process of CS-LBP for every image pixel is achieved using equation (9).

(9) $$\begin{array}{c} {CS\_LBP}_{\;R,D,T}\left(X,Y\right)=\overset{\left(D/2\right)-1}{\underset{i=0}{\sum }}M\left(d_{i}-d_{i+\left(D/2\right)}\right)2^{i}\text{,} \end{array}$$

$M\left(d_{i}-d_{i+\left(D/2\right)}\right)2^{i}=\left\{\begin{array}{c} 1,if\;x\geq T\\ \;0,if\;x<T \end{array}\right.$ Where, *d*_*i*_ and *d*_*i*+(*D*/2)_ are the values of the center-symmetric pair. *D* is the number of adjacent elements (size of 8 is used), *R* is the radius (*R*=2) of uniformly spared pixels on a circle and *T* is the threshold value (0.1). The reason for using a small threshold value is that it obtains robustness on flat image regions.

#### 4.4.4. Shape-Based Features/Geometric Analysis

The masses are categorized according to their shapes, sizes, distortions, and margins (borders) and are compared based on BI-RADS (Breast Imaging Reporting and Data System) [41]. To illustrate whether a tumor is benign or malignant. The shapes are usually oval, round, irregular lobular, or architecturally distorted in appearance. Tumors having round and oval shapes are usually benign. The tumors having irregular shapes exhibit the likelihood of malignancy. The margins of masses are micro-lobulated circumscribed, ill-defined, speculated, and obscured [42]. Shape features can explain certain characteristics of the geometry of a specific feature. Various regional descriptors are used to show the attributes of segmented masses [23]. These are area, eccentricity, solidity, perimeter, major axis length, convex area, and minor axis length [43].

**Figure 4:**
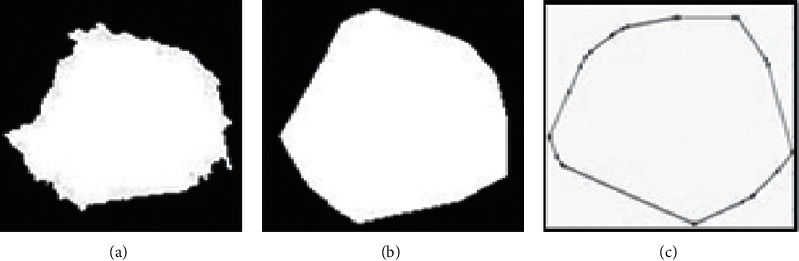
Region of interest. (a) Area. (b) Perimeter. (c) Convex hull.

**Figure 5:**
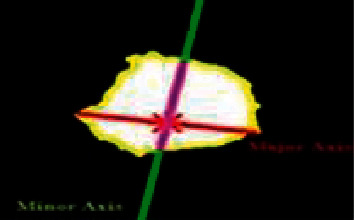
Minor axis and major axis.

*Area*: The Area of a region is the number of pixels in the object.
*Convex Area*: The convex hull or convex envelope area encloses the object depicted in Figure 4.
*Perimeter*: It is the number of pixels in the shape's boundary. If *v*_1,_ *v*_2,_ *v*_3,_.  … .., *v*_*N*_ is an edge, its perimeter is formulated in equation (10).
  (10) $$\begin{array}{c} Perimeter=\sum_{i=1}^{N-1}d_{i}=\sum_{i=1}^{N-1}\left|v_{i}-v_{i+1}\right|\text{.} \end{array}$$
*Major Axis length*: The major axis is the (*x*, *y*) endpoints of the lengthiest line that can be sketched across the shape. The major axis length of a shape is the pixel distance between the major axis endpoints portrayed in Figure 5 and is calculated by the formula as shown in equation (11).
  (11) $$\begin{array}{c} Major\;axis\;length=\sqrt{{\left(x_{2}-x_{1}\right)}^{2}+{\left(y_{2}-y_{1}\right)}^{2}}\text{.} \end{array}$$
*Minor Axis length*: The minor axis is the (*x*, *y*) endpoints of the lengthiest line that can be sketched through the shape while remaining perpendicular to the major axis.
*Eccentricity*: The minor axis length is the ratio of the major axis length of a shape. The outcome measures object eccentricity, which ranges between 0 and 1. Eccentricity is also called ellipticity.
  (12) $$\begin{array}{c} Eccentricity=\frac{{axislength}_{short}}{{axislength}_{long}}\text{.} \end{array}$$
*Solidity*: It calculates the density of an object. Solidity can be achieved as the ratio of an object's area to the convex hull area of the shape.
  (13) $$\begin{array}{c} Solidity=\frac{area}{convex\;area}\text{.} \end{array}$$

### 4.5. Classification

**Figure 6:**
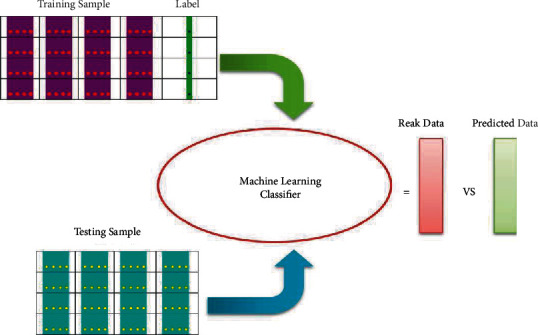
General framework of the classification process.

In this stage, a supervised classification technique is employed to differentiate the objects of concern based on features into an exceptional or normal class. The architecture of the classification process for training and testing is presented in Figure 6. Based on First and Second-Order Statistics Features, HOG, CS-LBP, and shape-based, the entity is categorized into two classes: benign (0) and malignant (1). The features are assessed to perform a classification by applying Linear Discriminant Analysis (LDA), Support Vector Machine (SVM), Ensemble, Decision Tree (DT), and K-Nearest Neighbor (KNN) classifier to distinguish the cancerous lesions from the benign ones. The K-fold method is chosen for cross-validation to avoid overfitting the classification in the suggested approach. We used 10-fold cross-validation for classifiers evaluation [44].

#### 4.5.1. Classification Algorithms

A classifier is an algorithm that has to be trained using labels to distinguish new unlabeled data among a fixed set of classes. Therefore, selecting a classifier is a significant step for the breast tumor detection system.*(1) K-Nearest Neighbor (KNN*): It is a nonparametric technique that considers all available observations and separates new observations based on their resemblance. The neighbors are chosen from a set of observations for which the exact class is identified. Next, the unknown observation is allocated to the utmost common class among its KNN through distance function. Finally, the Euclidean distance is calculated between the known and unknown cases using equation (14) [45].

(14) $$\begin{array}{c} d=\sqrt{\overset{K}{\underset{j=1}{\sum }}{\left(u_{j}-v_{j}\right)}^{2}}\text{,} \end{array}$$

where *u* and *v* are instances and *K* is a constant value defined by the user. In KNN, a sample is classified by a majority vote to attain a suitable class for a given k-values. KNN is easy to implement but vulnerable to irrelevant or redundant features.*(2) Support Vector Machine (SVM)*: It is a kernel-based supervised learning technique employed for regression and classification. SVM provides fast learning capability on large feature sets [46]. The differentiation is performed on a hyper-plane (line) design that splits the true and false training cases with a maximum margin to minimize the error in the multidimensional space. The training cases that define the line are support vectors [47]. The line can be computed as presented in equation (15).

(15) $$\begin{array}{c} W.r+v=0\text{,} \end{array}$$

where *r* indicates the input vector,*v* is the model's bias, *W* is termed the weight vector and expressed as*W*_1_, *W*_2_,…, *W*_*n*_.*(3) Decision Tree*:Decision trees classify the instances through a top-down approach by organizing them based on feature values. Each node signifies a case to be categorized and each branch designates a feature of that instance.*(4) Linear Discriminant Analysis (LDA)*: LDA is a classification technique developed by R. A. Fisher [48]. LDA finds the linear arrangement of the features that distinguish two or more categories of the data more accurately. In LDA, scatter matrices are used to find the efficacy of the classification: between-class and within-class. In addition, it determines the discriminant dimension in which the variance proportion of within-class and between-class is maximized [49].*(5) Ensemble Classifier*: It employs multiple learning methods to improve performance than any single method.

#### 4.5.2. Performance Measure of Classification

The proposed architecture is assessed through 109 ROIs images of the MIAS database using statistical parameters: accuracy, Matthews's correlation coefficient (MCC), sensitivity, and specificity to measure the performance of classification results.

Accuracy is the amount of true negative (TN) and true positive (TP) values among the total number of samples [50], as stated in equation (16).

(16) $$\begin{array}{c} Accuracy=\frac{TP+TN}{\left(TP+TN+FP+FN\right)}\text{.} \end{array}$$

Sensitivity is the proportion of positive samples correctly detected by the classifier, as shown by equation (17).

(17) $$\begin{array}{c} Sensitivity=\frac{TP}{\left(TP+FN\right)}\text{.} \end{array}$$

Specificity is the number of negative cases accurately identified by the classifier and formulated in equation (18).

(18) $$\begin{array}{c} Specificity=\frac{TN}{\left(TN+FP\right)}\text{.} \end{array}$$

MCC is an amount of the brilliance of two class orders and is demonstrated in equation (19).

(19) $$\begin{array}{c} MCC=\frac{TP\;x\;TN-FP\;x\;FN}{\sqrt{\left(\left(TP+FN\right)\left(TP+FP\right)\left(TN+FN\right)\left(TN+FP\right)\right)}}\text{.} \end{array}$$

Kappa statistic (*κ*) is the measurement of information applied to compute the understanding among ground truth and classification results [51]. The state of *κ* is presented in equation (20).

(20) $$\begin{array}{c} \kappa =\frac{\left(g_{0}-g_{e}\right)}{1-g_{e}}\text{,} \end{array}$$

Where *g*_0_is the experiential understanding between the ground truth and classification results, and *g*_*e*_ is the approximate likelihood of agreement, employing the relative information to determine each class's likelihood.

## 5. Results and Discussion

For experimental purposes, the above-mentioned statistical measures are used to accomplish the goodness of the proposed architecture. In addition, these features have been used to classify mammography images. SVM, KNN, Decision Tree, LDA, and ensemble classifiers have been utilized for classification.

### 5.1. Performance Measure of First and Second-Order Statistics Features

The performance results are displayed in Table 2. These values indicate that first and second-order statistics features can isolate two instances with remarkable accuracy (=94.5%) through the SVM classifier. Furthermore, a better rate of sensitivity (=94.1%) and specificity (=94.8%) ascertain that intensity histogram features can accurately isolate malignant and normal cases.

### 5.2. Performance Measure of HOG Features

The performance results of several classifiers for HOG features are exhibited in Table 3. These values indicate that HOG features yield an accuracy of 95.4% for mammographic images using the Ensemble classifier. Furthermore, HOG features achieve excellent sensitivity values (=96%) and specificity (=94.8%), demonstrating that HOG features can equally identify normal and malignant cases.

### 5.3. Performance Measure of CS-LBP Feature Sets

In this portion, an illustration of the assessment of CS-LBP features has been used. The performance is evaluated using SVM, KNN, decision tree, LDA, and ensemble classifier.  Table 4 presents classification accuracy for CS-LBP features.

### 5.4. Performance Measure of Shape Features

**Figure 7:**
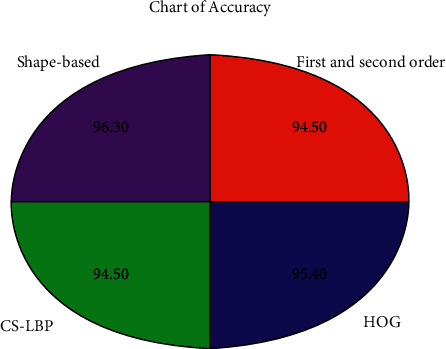
Comparison of classifiers performance.

The classification performance of shape features using KNN, SVM, Decision Tree, LDA, and Ensemble classifier is displayed in Table 4. These values specify that shape-based features obtained an accuracy of 96.3%. Such accuracy is marginally higher than that of First and Second-Order Statistics (=94.5%), HOG (=95.4%), and CS-LBP (=94.5%) as shown in Figure 7. Furthermore, shape-based features achieve excellent sensitivity values (=94.1%) and specificity (=98.2%), revealing that they can accurately distinguish between malignant and normal cases. Further, higher MCC values (=92.68%) demonstrate that they have high decency for classifying mammography images as delineated in Table 5.

### 5.5. Statistical Examination of Features against Classifiers

This part assesses the classification results' effectiveness and ground truth of the related features by Kappa statistics [51]. The classification performance for proposed features has been demonstrated in Table 6. These feature sets display amazing performance achieved via kappa statistics employing SVM, KNN, and ensemble classifier. Furthermore, the Kappa statistic of shape-based features through the ensemble classifier is 0.92, demonstrating nearly a perfect agreement with the ground truth.

In the proposed CAD system for the mobile unit, all the steps are performed in a sequence without manual intervention. Various existing methods for tumor detection and their performance results are provided in Table 7. Some common results of the suggested strategy are also compared with existing ones.

## 6. Conclusions and Future Work

In this research, the IoMT mammography CAD system's architecture for identifying breast abnormalities has been presented. The proposed model used the concept of IoMT with the help of cloud computing. The processing of the CAD system is completed locally, and the decision of the CAD system and the mammography images are uploaded to the cloud for further assessment by the radiologist. Deployment of such a system in remote areas will facilitate the patient to start her treatment immediately. The proposed CAD system employs the region-growing algorithm to segment mammograms. After tumor segmentation first and second-order statistics, HOG, CS-LBP, and shape-based features are extracted. These features are fused into a feature vector to classify masses as benign or cancerous using different algorithms, including Decision Tree, SVM, KNN, LDA, and ensemble classifier. A noteworthy accuracy of 96.3% is obtained with geometrical analysis on mammogram images of the MIAS dataset. The result portrays that shape-based features improve classification accuracy, sensitivity, and specificity through ensemble classifiers. The proposed strategy has also been compared with previous mammogram tumor detection methods. Higher classification accuracy is observed as a result of this study. The main limitation of this research work is the use of conventional machine learning methods. Future research may focus on using deep learning, which is recently used in diagnostics and predictions. Moreover, classifying mammogram images into more than two classes corresponding to BI- RADS level is also a good research direction.

## Figures and Tables

**Table 1:** Summary of related work on breast cancer diagnosis.

Ref.	Dataset	Classifiers	Accuracy (%)
[2]	WBCD	ANN	98.68
[18]	*S*-WAVE	Random forest and support vector machine.	95
[19]	MIAS	CNN models	90.47
[20]	—	YOLO and RetinaNet models	91
[21]	MIAS	UNet (DenseNet) with attention gates (AG)	78.38
[22]	—	Naive bayesian and artificial neural networks	98
[23]	OASBUD	Decision tree, KNN,	97

**Table 2:** Performance-based on first and second-order statistics.

Classifiers	Accuracy %	Sensitivity %	Specificity %	MCC %
Decision tree	88.1	86.27	89.66	76.03
LDA	53.2	37.25	67.24	4.71
SVM	94.5	94.12	94.83	88.95
KNN	93.6	90.20	96.55	87.19
Ensemble	92.7	90.20	96.55	87.18

**Table 3:** Performance-based on HOG features.

Classifiers	Accuracy %	Sensitivity %	Specificity %	MCC %
Decision tree	93.6	96.08	91.38	87.28
LDA	72.5	70.59	74.14	44.73
SVM	94.5	88.24	100	89.42
KNN	94.5	92.16	96.55	88.98
Ensemble	95.4	96.08	94.83	90.81

**Table 4:** Performance-based on CS-LBP features.

Classifiers	Accuracy %	Sensitivity %	Specificity %	MCC %
Decision tree	88.1	86.27	89.66	76.03
LDA	53.2	37.25	67.24	4.71
SVM	94.5	94.12	94.83	88.95
KNN	93.6	90.20	96.55	87.19
Ensemble	92.7	90.20	96.55	87.18

**Table 5:** Performance-based on shape features.

Classifiers	Accuracy %	Sensitivity %	Specificity %	MCC %
Decision tree	92.7	84.31	100	86.08
LDA	60.6	56.86	63.79	20.68
SVM	94.5	88.24	100	89.42
KNN	95.4	94.12	96.55	90.79
Ensemble	96.3	94.12	98.28	92.68

**Table 6:** Kappa statistics.

Features	DT	SVM	KNN	Ensemble
First and second-order statistics	0.68	0.58	0.83	0.87
HOG	0.86	0.91	0.88	0.90
CS-LBP	0.76	0.88	0.87	0.87
Shape-based	0.85	0.88	0.90	0.92

**Table 7:** Comparing with some existing methods.

Ref.	Dataset (MIAS)	Classifiers	Accuracy %
Proposed method		Ensemble	96.3
[52]		Ensemble	89.5
[53]		SVM	94
[54]		SVM	91.37

## Data Availability

The data that support the findings of this study are available on request from the authors.

## Abbreviations

ACS: American cancer society
BI-RADS: Breast imaging reporting and data system
CAD: Computer-aided diagnosis
CLBP: Completed local binary pattern
CS-LBP: Center-symmetric local binary pattern
DDSM: Digital database for screening mammography
DT: Decision tree
ECG: Electrocardiogram
FN: False negative
FP: False positive
GLCM: Gray-level co-occurrence matrix
HOG: Histogram of oriented gradients
IoMT: Internet of medical things
KNN: K-nearest neighbor
LBPV: Local binary pattern variance
LDA: Linear discriminant analysis
LPQ: Local phase quantization
LTP: Local ternary pattern
MCC: Matthews correlation coefficient
MIAS: Mammographic image analysis society
MIAS: Mammographic image analysis society
MLP: Multilayer perceptron
OWBPE: Opposite weight back propagation per epoch
RLTP: Radial local ternary patterns
ROI: Region of interest
SCBDL: Soft clustered based direct learning
SD: Standard deviation
SIFT: Scale-invariant feature transform
SVM: Support vector machine
TN: True negative
TP: True positive.
